# Converging evidence for the pro-inflammatory role of p-glycoprotein in Th2 polarized chronic rhinosinusitis endotypes

**DOI:** 10.1186/2045-7022-5-S4-P7

**Published:** 2015-06-26

**Authors:** Benjamin Bleier

**Affiliations:** 1 Massachusetts Eye and Ear Infirmary, Harvard Medical School, Boston, USA

## Background

T-helper 2 (Th2) inflammation is a hallmark of Chronic Rhinosinusitis with Nasal Polyps (CRSwNP) and eosinophilic CRS (eCRS) although the pathogenesis is poorly understood. P-glycoprotein (P-gp) is an efflux pump which is expressed within sinonasal mucosa and is capable of regulating cytokine transport in both epithelial and T-cells. The purpose of this study was to synthesize multiple lines of evidence suggesting that P-gp may promote inflammation in Th2 polarized CRS endotypes.

## Methods

The findings of 8 prior studies (5 in vitro and 3 clinical) by our research consortium were examined for the presence of a consistent mechanistic framework linking the presence of altered P-gp expression and function to enhanced Th2 inflammation.

## Results

Two studies performed in primary epithelial cell culture demonstrated that P-gp expression is responsive to TLR4 stimulation and modulates Th2 cytokine secretion. These findings were replicated in 2 organotypic nasal polyp explant studies which demonstrated that the secretion of IL-5 and TSLP was highly correlated with P-gp expression and could be blocked using the P-gp inhibitors Verapamil or Zosuquidar. Three complimentary clinical studies demonstrated that P-gp was specifically overexpressed in polyp tissue and that expression correlated with tissue eosinophilia, Lund-Mackay Scores, and Kennedy Osteitis scores. A final in vitro study demonstrated that Clarithromycin and Itraconazole possess occult P-gp inhibitory properties.

## Conclusion

Multiple lines of evidence suggest that P-gp regulates the secretion of Th2 polarizing cytokines from its host cell. Furthermore, the concentration of secretion is strongly correlated with the level of P-gp expression indicating a direct pro-secretory role. The P-gp overexpression found in CRSwNP and eCRS may therefore be interpreted as playing a central role in the maintenance of Th2 inflammation through enhanced cytokine secretion. The anti-P-gp effects of Clarithromycin and Itraconazole, two drugs with known anti-inflammatory properties, hint at a possible novel therapeutic strategy mediated by P-gp inhibition.

